# Sexual and reproductive health and rights knowledge, perceptions, and experiences of adolescent learners from three South African townships: qualitative findings from the Girls Achieve Power (GAP Year) Trial

**DOI:** 10.12688/gatesopenres.13588.1

**Published:** 2022-05-03

**Authors:** Melanie Pleaner, Cecilia Milford, Alison Kutywayo, Nicolette Naidoo, Saiqa Mullick

**Affiliations:** 1 Wits Reproductive Health and HIV Institute (Wits RHI), Faculty of Health Sciences, University of the Witwatersrand, Johannesburg, Gauteng, 2193, South Africa; 2 MRU (MatCH Research Unit), Department of Obstetrics and Gynaecology, University of the Witwatersrand, Durban, Kwa zulu Natal, 4001, South Africa

**Keywords:** Adolescents, sexual and reproductive health, rights, transactional sex, contraceptive choices, South Africa

## Abstract

**Background: **Adolescence is a time of psycho-social and physiological changes, with increased associated health risks including vulnerability to pregnancy, HIV, sexually transmitted infections, and gender-based violence. Adolescent learners, from three townships in South Africa, participated in a 44 session, after-school asset-building intervention (GAP Year), over 2 years providing sexual and reproductive health (SRH) education. This paper explores adolescent learners’ SRH, sexual risk and rights knowledge; perceptions about transactional sex; and contraceptive method preferences and decision-making practices.

**Methods:** The intervention was conducted in 13 secondary schools across Khayelitsha, Thembisa, and Soweto, South Africa. A baseline survey collected socio-demographic data prior to the intervention. Overall, 26 focus group discussions (FGDs): 13 male and 13 female learner groups, purposively selected from schools, after the intervention (2 years after baseline data collection). Descriptive analyses were conducted on baseline data. Qualitative data were thematically coded, and NVivo was used for data analysis.

**Results:** In total, 194 learners participated in the FGDs. Mean age at baseline was 13.7 years (standard deviation 0.91). Participants acquired SRH and rights knowledge during the GAP Year intervention. Although transactional sex was viewed as risky, some relationships were deemed beneficial and necessary for material gain. Negative healthcare provider attitudes were the main barrier to healthcare service utilisation. There was awareness about the benefits of contraceptives, but some myths about method use. The injectable was the preferred contraceptive method, followed by the implant, with equal preference for condoms and oral pill.

**Conclusions** *: *An afterschool intervention at school is a viable model for the provision of SRH and rights education to learners. Recommendations include the need for risk reduction strategies in the curriculum, dealing with misconceptions, and the promotion of informed decision making. Endeavours to ensure health services are youth friendly is a priority to limit barriers to accessing these services.

## Introduction

Adolescence is a time of dynamic psycho-social, physiological and sexual change, accompanied by an increase in health-related risks ^1^. Understanding this for young people in sub-Saharan Africa is particularly important where approximately 3 out of 10 unmarried adolescents have ever had sex ^2^, and more than 50% of rural adolescent girls and young women (AGYW) (15–24 years of age) are estimated to have been pregnant before the age of 18 ^3^. With an increased vulnerability to early and unintended pregnancy, sexually transmitted infections (STIs), gender-based violence (GBV) and human immunodeficiency virus (HIV) ^4^, AGYW in sub-Saharan Africa account for one quarter of new HIV infections, even though they comprise only 10% of the population ^5^. Adolescent girls (aged 15–19 years) are particularly vulnerable, comprising 80% of all new HIV infections in sub-Saharan Africa, and twice as likely to get HIV than their male counterparts ^6^. This is driven by multiple cross cutting socio-economic and structural factors, including harmful gender norms and inequalities, social and cultural norms, transactional and age disparate sex, sexual and intimate partner violence, and poor access to social and health-related services ^7– 9^. Access to knowledge is also of concern – with HIV-knowledge in sub-Saharan Africa being low ^10^, for example only 36.4% and 29.8% of young men and women, respectively, were shown to have a basic knowledge about HIV prevention ^3^.

The situation in South Africa mirrors trends in sub-Saharan Africa. Young people, particularly AGYW, are disproportionally affected by HIV: young people (aged 15–24) represented more than a quarter of all new infections in 2017, and AGYW were three times more likely to be infected with HIV than their male counterparts ^11^. AGYW have a 1.5% incidence and 26.3% prevalence of HIV infection ^12^. A study in rural KwaZulu-Natal in South Africa, demonstrated high STI prevalence in 15–24 year olds – 14% of all had a curable STI (chlamydia, gonorrhoea, syphilis or trichomoniasis), and the prevalence of bacterial vaginosis was 41.1% in women aged 15–19 years ^13^. GBV rates are high in South Africa, with between 25–40% of women having reported experiencing sexual and/or physical intimate partner violence in their lifetime ^14^, and 17% of women receiving HIV treatment reported sexual violence in their lifetime ^15^. Due to the underreporting of violence, the true incidence of GBV is likely to be higher ^16, 17^.

Poverty and unemployment rates are high in South Africa. In the first quarter of 2021, 32.4% of 10.2 million young people (aged 15–24) were not in employment, education or training ^18^. Consequently, young people may be motivated to engage in transactional relationships with older partners for financial or material gain ^19, 20^, known as the “sugar daddy” or “sugar mommy” phenomenon ^21, 22^, and as “blessers” and “blessees” in South Africa ^23, 24^. Due to the inequitable power dynamics in these relationships, many young women may be subjected to GBV ^21^ and may not be able to negotiate condom use ^25, 26^, putting them at increased risk for unintended pregnancies, STIs and HIV acquisition ^20– 22^.

Adolescents face numerous barriers when accessing sexual and reproductive health (SRH) and, more specifically, contraceptive services. Adolescents in sub-Saharan Africa have unmet needs in relation to contraception ^27^. The main barriers facing young people accessing contraceptive services include fear of stigma, shame and embarrassment, lack of privacy, provider attitudes and lack of knowledge about services and contraceptive options ^28– 30^. There are also barriers relating to providers being judgemental, and gaps in their contraceptive knowledge, skills and training to respond to the specific needs of adolescents ^30, 31^. In South Africa, there are several initiatives and programmes to dismantle barriers and standardise the package of adolescent and youth friendly services ^32– 34^. This is supported by government guidelines which emphasise the importance of accessible and acceptable services for young people ^35– 38^. However, although these guidelines exist, implementation at facility level has been challenging, and service provision is not always youth friendly ^33^.

Adolescent contraceptive use and knowledge is poor in Sub-Saharan Africa ^27^. Evidence from demographic health surveys (DHS) in sub-Saharan Africa showed that most adolescents (92.4%) (aged 15–19 years) reported no contraceptive use, although 21.6% reported recent sexual activity ^39^. A later review of DHS data reported that only 24.7% of AGYW (15–19 years) in 29 sub-Saharan African countries used modern contraceptives ^40^, with the majority preferring injectable contraceptives (39.9%) and oral pills (31.4%) ^39^. 

In South Africa more specifically, the most recent DHS in 2016 ^41^ showed that 60% of sexually active women were currently using a method of contraception, and 19% of sexually active women had an unmet need for contraception ^41^. The 2012 National HIV Prevalence, Incidence, and Behaviour Survey ^42^, found that about a third of women aged 15–19 were using modern contraception ^43^. Furthermore, 33.5% of women had reported a pregnancy in the last five years, and only a third of these had desired to be pregnant ^42^. Specifically, only 10.1% of women 15–19 years, and 20.9% of women aged 20–24 years had desired the pregnancy ^42^. Of those women who reported a pregnancy in the past five years, only 12.8% of 15–19 year olds, and 19.7% of 20–24 year olds, had been using contraception ^42^. In terms of method preference, injectables are the predominant method used, followed by oral contraceptive pills ^43^. The implant was introduced into South Africa in 2014, but only 4% of South African women were using it in 2016 ^38, 44^. Research on adolescents in South Africa (Limpopo Province) demonstrated that most did not have knowledge about the emergency contraceptive, intrauterine device (IUD) or female condom ^45^. Contraceptive preferences are shaped by choice, provider bias and training, demand creation, availability, misinformation and side effects ^46, 47^. Other factors affecting uptake and use are linked to pressure from male partners, fear of parental reaction, poor contraceptive education, and counselling ^45^. Therefore, ensuring contraceptive choice is critical for adolescents to have options when making contraceptive use decisions.

Age-appropriate sexuality education is seen as an important intervention for mitigating against risk in young adolescents. Early adolescence is seen as an ideal time to conduct education with a focus on gender and rights, before harmful gender norms become entrenched, enabling improved SRH and non-violent outcomes ^48^. School and curriculum based educational interventions have been implemented globally, seeking to improve adolescent SRH outcomes ^49^. This has largely been informed by a rigorous evidence review of over 77 randomised controlled trials and systematic reviews conducted by UNESCO ^50^, evaluating the impact of Comprehensive Sexuality Education (CSE). The findings clearly demonstrate that CSE produces favourable outcomes, specifically reducing risky sexual behaviours, sexual debut, number of sexual partners whilst improving health seeking behaviour, knowledge and uptake of preventative services such as contraception and condoms. Peer-led interventions have demonstrated moderate effectiveness at improving behavioural outcomes, such as increasing HIV-related knowledge and improving condom usage ^51^.

Young adolescence (10–14 years) in particular, is the time of onset of puberty and sexual maturation and is a transitional period that shapes later adolescent and adult health behaviours ^48, 52, 53^, including SRH behaviours which may cause diseases later in life ^10^. There is very little research on the SRH of this younger adolescent population, for both males and females. There is a need to focus on this missed and neglected age group to promote optimal SRH in future ^10^. Therefore, this research included younger adolescents (from 12 years of age) seeking to further understand their knowledge, experience and needs.

## Girls Achieve Power (GAP Year) Trial

Girls Achieve Power (GAP Year), a cluster randomised controlled trial (cRCT), was a social asset building intervention focussing on SRH, across 26 schools in Gauteng and the Western Cape Provinces in South Africa ^54^. It provided SRH information aligned to the CSE curriculum and sought to contribute to and complement the CSE programme ^55^. The schools were in three peri-urban townships: 14 schools in Khayelitsha (Western Cape Province), six schools in Soweto, and six schools in Thembisa (Gauteng Province). Schools were selected in collaboration with the Department of Basic Education using the following inclusion criteria: mixed sex public high schools in three townships; in quintiles 1–3 ^1^ which had not been exposed to any asset building interventions in the past six months. A one-to-one (1:1) random stratification scheme was employed, assigning each school to either intervention or control groups, with 13 schools in each study arm.

The GAP Year Trial tested the effectiveness of a four-pronged ecological intervention: a sports-based peer facilitated after-school asset-building intervention, a parent/guardian component, linkage to care, and school safety. The intervention is described in further detail in Kutywayo, *et al* ^54^. For the after-school intervention, a two-year curriculum (of 44 sessions in total) was designed and implemented, delivered after school by peer coaches, covering different aspects of SRH and rights, HIV, STIs, contraception, decision making, sex and gender. The trial aim was to reduce school dropout among adolescent girls between grades 8–10 and increase reporting of GBV. Complimenting these outcomes, the intervention sought to improve adolescent girls’ agency and safety while shifting gender attitudes and encouraging positive behavioural change among adolescent boys.

For this manuscript, we explore the SRH knowledge and behaviour, including risks faced, of grade 9 and 10 learners, from three townships in the Western Cape and Gauteng Provinces, who participated in this intervention. We focus on four thematic areas: adolescent learners’ knowledge about SRH and understanding of sexual risk, risk reduction and rights; perceptions related to transactional sex and age disparate relationships; health seeking behaviour and experiences; and contraceptive method preferences and decision-making practices.

## Methods

### Study design

A cross sectional qualitative study utilising data from single sex focus group discussions (FGDs) explored adolescents’ knowledge and experiences on health, SRH (including contraception knowledge and uptake) and risky behaviours. The FGDs were conducted after the two-year GAP Year intervention had been completed. No participant demographic information was collected during the FGDs. FGD participant socio-demographic and risky behaviour data was extracted from a baseline survey conducted with all GAP Year participants prior to the implementation of the GAP Year intervention ^56– 58^.

### Study setting and population

The qualitative study was conducted in all 13 intervention schools in the three South African peri-urban townships, Khayelitsha (7 schools), Soweto (3 schools) and Tembisa (3 schools).

All grade eight learners, at selected schools were eligible to participate in the baseline survey and the GAP Year intervention, irrespective of sex, age, or race. The study population for the FGDs consisted of grade 9 and 10 male and female learners who were enrolled in the GAP Year Trial and had participated in the two-year GAP year intervention. Stratified purposive sampling ^59^ was used to select learners for participation in the FGDs. Attendance data was extracted from the GAP Year intervention registers. Only those that had participated in a minimum of 32 of the 44 sessions of the two-year GAP Year intervention were invited to participate. Eligible participants were recruited face to face, by peer coaches, who went to the schools and invited them to participate in the FGDs. The sample size for the FGDs was between seven to ten participants, based on the average for this methodology ^60^. One female and one male FGD was conducted for each school in the intervention study arm.

FGDs were conducted either in GAP Year schools or at a local venue in the community. Participants were assigned a unique number, to ensure anonymity when identifying themselves for the audio recording. Prior to starting the FGD, participants were reminded that, due to the nature of FDGs, they were not anonymous, but participants were encouraged to keep what was discussed in the group confidential.

### Data collection

***Collection of demographics, health seeking and behavioural data.*** A baseline survey was conducted as part of the GAP Year trial with all GAP Year participants (see *Extended data* ^61^). Data for the first section (demographics, knowledge and attitudes) was collected by trained fieldworkers and captured directly onto an android tablet, formatted with the Research Electronic Data Capture (REDCap, RRID:SCR_003445) system ^62^, lasting 45 minutes to 1 hour. The second, a behavioural section, lasted 20–30 minutes and was administered using the audio computer assisted self-interviewing (ACASI) method, seeking to reduce social desirability, because of the sensitive nature of the questions. For the purposes of this manuscript, key variables from the baseline survey have been extracted for the FGD participants, to explore their background and demographic characteristics and contextualise their qualitative discussions.

***Collection of FGD data.*** A total of 26 FGDs were conducted, with 13 male and 13 female groups, respectively. Semi-structured FGD guides collected data based on the content of the GAP Year intervention facilitators’ manuals (see *Extended data* ^63^). FGDs were conducted in isiXhosa, isiZulu, Sesotho or Sepedi, the different local languages, as was preferred by the participants. They were between 45 minutes and an hour in duration. Data saturation was reached, and no more FGDs were conducted after these.

FGDs were conducted in 2019 and 2020 and were facilitated by one experienced researcher (facilitator) and assistants. The facilitators were Black South Africans with master’s degrees. The assistants were coach mentors and assisted the facilitator with logistics and note taking. Facilitators and assistants were trained in research ethics, qualitative research methods and the study protocol, and were proficient in the local languages spoken at the study sites. Female facilitators and assistants conducted the female learner FGDs, and the male learner FGDs had a combination/variation of male and female facilitators and assistants. There were no existing relationships between facilitators and participants prior to data collection. Learners were informed of the aims of the data collection and that the researchers were part of the research team. Immediately following each FGD, the researcher and assistant completed field notes and observations of the FGD.

### Data management and analysis

The completed baseline surveys were stored on encrypted password-protected tablets and the synced data was stored on secured organisational servers. All data from the REDCap ^62^ and ACASI systems were exported into Stata 17 (RRID:SCR_012763) ^64^, and descriptive analyses were conducted to describe the socio-demographic variables of the FGD participants.

The FGDs were transcribed and translated directly into English. Three coders (a researcher, a research assistant, and an external consultant) generated codes for male and female learners iteratively, based on input from the questions in the guides as well as from emergent themes from the data. Data were double coded to ensure reliability of coding. Data coding and analysis was facilitated using NVivo 12 (QSR International, RRID:SCR_014802) software ^65^. A deductive approach to data analysis was used in this study. 

## Ethical approval and considerations

The University of the Witwatersrand’s Human Research Ethics Committee (HREC) approved the GAP Year trial (#M160940) in October 2016. FGDs formed part of the trial and were included in the ethics approval from the onset. The provincial research committees at the Western Cape and Gauteng Department of Basic Education, and each participating school also provided written approval. Parents or guardians provided written informed consent and written assent was provided by participating learners for participation in the FGD and for the FGD to be audio recorded. The participating schools, parents and learners were fully informed about the voluntary nature of participation in the study, and of confidentiality of data management.

## Results

### Demographics

Demographic details were collected at baseline, approximately two years prior to conducting the FGDs ( Table 1), therefore all details presented in the table are representative of baseline, prior to the intervention and two years prior to the FGDs. Of the 194 who participated in the FGDs, 38.2% (n=47) were female, 61.8% (n=76) male, and there were missing data for 71 participants ^63^. In total, 78% (n=96) were aged 12–14 years, with the mean age being 13.7 years at baseline (acknowledging the natural aging of the cohort for this study). The majority were Black African (99.2%, n=122). At baseline, 22.8% (n=28) had ever had sex: of those, 89.3% (n=25) had had their sexual debut at 14 years or under. At baseline, 20.3% had ever used contraception (n=25).

**Table 1.** Descriptive demographics of focus group discussion (FGD) participants (N = 194), using baseline data.

Variables	Total		Sex			
			Female		Male	
	%	N	%	N	%	N
***Sex***						
Female	38.2%	47	-	-	-	-
Male	61.8%	76	-	-	-	-
**Total**	100.0%	123	-	-	-	-
*Missing*	*71*					
***Age category***						
12–14 years	78.0%	96	80.9%	38	76.3%	58
15–18 years	22.0%	27	19.1%	9	23.7%	18
**Total**	100.0%	123	100.0%	47	100.0%	76
*Missing*	*71*					
*Mean age*	13.7 [SD: 0.91]		13.8 [SD: 0.85]		13.7 [SD: 0.92]	
***Race***						
Black African	99.2%	122	97.9%	46	100.0%	76
Coloured	0.8%	1	2.1%	1	0.0%	0
**Total**	100.0%	123	100.0%	47	100.0%	76
*Missing*	*71*					
***Ever had sex***	22.8%	28	4.3%	2	34.2%	26
Refused to answer	5.7%	7	0.0%	0	9.2%	7
*Missing*	*71*					
***Age of first sexual intercourse (n=28)***						
14 years and under	89.3%	25	50.0%	1	92.3%	24
15–17 years	10.7%	3	50.0%	1	7.7%	2
**Total**	100.0%	28	100.0%	2	100.0%	26
***Ever used contraception***	20.3%	25	14.9%	7	23.7%	18
*Missing*	*71*					

FGD results are grouped into the following four thematic areas which will be unpacked in turn: adolescent learners’ knowledge about SRH and understanding of sexual risk, risk reduction and rights; perceptions related to transactional sex and age disparate relationships; health seeking behaviour and experiences; and contraceptive method preferences and decision-making practices.

### SRH knowledge and understanding of sexual risk, risk reduction and rights

***Exploring SRH knowledge.*** Male and female learners reported that they had learnt about both male and female condom use, describing how they learnt about the purpose of condoms - for the prevention of pregnancy, HIV and STIs (and dual protection). Some also highlighted that they were taught about how to use condoms correctly, to check for condom quality (e.g. expiry dates, tears), and appropriate disposal of condoms.

*P08: “We learnt about how to use a condom... they showed us how to use male and female condoms during condom demonstration”. (Khayelitsha, Group 5, females)*
*P07: “Most people did not know that condoms protect against not only HIV, but also other STIs". (Khayelitsha, Group 3, males)*
*P04: “I leant that when sleeping with a girl you might get a disease without using a condom or they could get pregnant”. (Soweto, Group 3, males)*
*P04: “Whenever I used a condom, I never used to check the expiry date but now I know that I should check whether it is fine or not”. (Tembisa, Group 1, males)*

Participants had also learnt about different ways to prevent pregnancy and the benefits of doing so.

*P04: “Condom can prevent pregnancy and illnesses”. […]* *P01: “What I know is that you should not only use a condom; you must protect yourself by taking pills to prevent getting pregnant”. […]* *P02: “We were taught how to prevent pregnancy. Like when you have had unsafe sex, the consequences are that you can become pregnant and become a young mother and you must leave school and work for your child. We were also told about different contraceptives and how they work. For instance, if you start taking the (oral contraceptive) pill today; you will be protected after 7 days and if you follow the instructions correctly you will not fall pregnant”. (Khayelitsha, Group 6, females)*

Many participants, both male and female, reported learning about STIs and HIV during the GAP Year intervention. Some noted they had learnt more about HIV transmission than they had previously known.

*P05: “When I got to GAP Year, I knew some things because there is free wi-fi for 15 minutes at this place where I used to go and search for information on the internet. Like I knew about STIs, but I did not know that there were illnesses that fall under it, like syphilis and gonorrhoea illnesses for down there”. (Khayelitsha, Group 3, males)*
*P07: “After joining GAP Year, I learnt that HIV is not only transmitted through sex. You can contract it if someone has a cut and encounters blood of someone who is HIV. Also, you can be born with it from your mother”. (Khayelitsha, Group 2, males)*

Some participants discussed learning about HIV prevention strategies such as condom use and circumcision, and females in one group talked about learning about pre- and post-exposure prophylaxis (PrEP and PEP).

*P03: “What I learnt from the coaches is that when you are circumcised the rate of getting infected is less”. (Soweto, Group 3, males)*
*P07: “I learnt about PEP and PrEP […] PEP is a pill that prevents after sexual intercourse and PrEP is for before you have sexual intercourse […] It helps [prevent] you from contracting HIV”. (Soweto, Group 2, females)*

Sexual behaviour, including rights, risk reduction strategies and respect – including understanding consent to sex, were all described as information learnt by both male and female respondents in some groups.

*P01: “I learnt to accept. When someone says no it is a no. […] When someone says no it is a no”*. *P03: “I was taught to accept the person I am without the pressures of other people and not compare myself to others”. […]* *P02: “I am adding to what number one said, I should not force a female to have sex but rather wait for her until she is ready to have sex”. […]* *P01: “To abstain and that maybe when your girlfriend wants to have sex, you just say no. And say that you are young without any pressure”. (Tembisa, Group 2, males)*
*P07: “So that’s what I learnt that maybe if we grow up and decide to have partners, we should have only one partner”. (Tembisa, Group 2, females)*
*P02: “Like after having sex with a female, I used to tell her ‘just go home I am done with you’. But now, I know that it is not right to do so”. (Tembisa, Group 1, males)*

***Exploring understandings of risk related to sex.*** Participants were asked to describe their understandings of risk related to sex. Overall, males and females had similar understandings of risk related to sex, with variations in depth of knowledge.

The majority described sex without using a condom or without protection, as ‘‘risky’’ sex. Most groups elaborated that this could lead to risk of HIV infection, and some also noted that it could result in pregnancy. Several females mentioned that risky sex could lead to STIs, but no males talked about the risk of STI acquisition.

*P04: “Yes, coach, it is having sex without a condom, which result to unplanned pregnancy, and you can also contract HIV”. (Soweto, Group 2, males)*
*P09: “When you sleep with someone only to find out that they have HIV or STDs and fall pregnant and are unable to finish school, and the males is also in school so he can’t afford to pay for the baby”. (Soweto, Group 2, females)*

Having multiple partners or being unfaithful was described as risky by some females and males. Some males further described risky sex as having sex with an HIV-positive person, or knowingly being HIV-positive and having sex with others. Some females felt that not knowing a partners’ HIV status was risky.

*P07: “Being at risk it means like being in danger, so I can say that if maybe a person knows maybe that the partner is not faithful and then she decides not to use protection, so that is risky because that person, number one she can get uh STD diseases, and she might also get umm pregnancy and they check this”. (Tembisa, Group 2, females)*
*P01: “Maybe it is having sex while knowing that person has HIV”. (Tembisa, Group 2, males)*
*PID unknown: “Risk, I think it refers to having sex knowing you are HIV and you end up infecting someone”. (Khayelitsha, Group 5, males)*
*P02: “Like sleeping with your male friend without using a condom while you do not know his status”. (Khayelitsha, Group 6, females)*

Sexual rights were deemed important, and sex without consent was raised as a risky sex behaviour by some females.

*P09: “Something like going out with someone to drink and you have a black out then he sleeps with you without your consent”. (Khayelitsha, Group 7, females)*

### Transactional sex and age disparate relationships

Participants were asked to describe blessers or sugar mommies. Most described them as older, married people with money:

*P02: “[Blessers are] People with money, they want things from you”. (Khayelitsha, Group 1, males)*
*P01: “Your mom has a friend and she come to your house coming to check you out and you meet up with her”. (Khayelitsha, Group 3, males)*

***Reasons for transactional sexual and age disparate relationships.*** Participants described that young people date blessers/sugar mommies/mamas largely for gifts or money for various reasons. Sometimes these relationships were deemed necessary for survival. Some adolescents from poor families used the money/goods from these transactional relationships to support their families. In a few cases, learners could get support for school fees through these relationships.

*P08: “Like the reason why like females go to blessers some like its family background, you find that there is nothing, you find that she is a bread winner and like there is not easy way for her to get money or something like. She only thinks of getting someone I will be able to look after my family”. (Tembisa, Group 3, females)*
*P10: “Some sugar mamas even pay for your school fees and buy you things that you need for school”. (Khayelitsha, Group 7, males)*

Others engaged in these relationships for material gain, improved lifestyles, and as a result of peer pressure. It was felt that females are influenced to date these older men in exchange for money, better clothes, shoes and hairstyles, and that males date older women in exchange for money, better clothes and opportunities to drive nice cars and stay in nice houses. Participants described how in some cases these desires were influenced by peer pressure.

*P06: “Friends influencing you; like saying ‘friend, they bought me something at home, (carvela) shoes’. Then you think to yourself, I also want it. You end up dating older men because you want him to give you things that your friends have”. (Khayelitsha, Group 3, females)*
*P04: “To be seen in the community. Like having a car and giving me money, so the lady will ask for sex in exchange of those things”. (Tembisa, Group 2, males)*

A few female participants felt that some young females date blessers as an act of rebellion towards their parents (who may be absent/abusers), or because they are lacking love and support at home.

*P07: “[T]he reason other females deal with blessers is because of like anger she has. And even the way her friends guide her. [….] Like some we have mothers that are abusing substances. […] So like it gives you that thing of saying what’s the use of being a good person while I know my mother is doing such things? So I look for someone who does everything for me, someone who makes me happy”. (Tembisa, Group 3, females)*
*P04: “Because they have not received a father’s love, you see. So, they will go there; or maybe at home they do not afford a lot of things”. [….]*
*P02: “For a female, if they are not loved at home, if they see an older person that loves them, they go for them”. (Khayelitsha, Group 3, females)*

Male and female respondents both reported that some young males date sugar mamas for sexual experience that they are unable to get from younger females.

*P02: “Males will say, ‘females are too young, with no experience’. So, they go for an older person with experience”. (Khayelitsha, Group 6, females)*

***Community acceptability of transactional sexual relationships.*** Participants largely agreed that transactional sexual relationships were not acceptable in their communities. However, some felt that even if they were not viewed as acceptable, they still occurred.

*P08: “It is not right in the community. But if you are old enough and it is working for you, then go for it. If she provides and everything is fine, I do not see a problem. You should not care about what other people say whether it is working out or not”. (Khayelitsha, Group 7, males)*

One group of males from Khayelitsha, however, felt that the community might accept these relationships, as they provide a source of income for the family. Furthermore, a group of females from Khayelitsha suggested that parents may even support these types of relationships, especially when they benefit the family.

*F: “Is the idea of sugar mamas and sugar daddies acceptable in your community?” […]*
*P03: “It depends. I will tell you about something that happens; if you are a 16 year old, and you date a person who has grandchildren, the community does not say anything because you will be having money”. (Khayelitsha, Group 3, males)*
*P02: “In other communities, they are accepted but in others, they are not. Some parents will say, ‘my child go date that person because he has money and he will take care of us’”. (Khayelitsha, Group 6, females)*

***Consequences of age disparate and transactional sexual relationships.*** In spite of the perceived benefits of having a blesser/sugar mama (described above), adolescent males and females also discussed various negative consequences of being in these types of relationships. The most common concerns were related to the unequal power in these relationships, where adolescents could be controlled and forced to do things they didn’t want to do.

*P03: “I think blesser is going to control you, he will tell you what to do and what not to do”. […]*
*P05: “I think the blessers are controlling to do something you don’t want, yah”. (Tembisa, Group 2, females)*

For example, there were concerns that blessers/sugar mamas could insist on unprotected sex, resulting in transmission of HIV, STIs and unplanned pregnancies – which in some cases could lead to abortions.

*P03: “This person is old. So, they want to sleep with you, and she has power over you. She tells you not to use a condom and you end up getting HIV”. (Khayelitsha, Group 7, males)*
*F: “What do you think people get from blessers?” […]*
*P03: “They get STIs and HIV; then they end up dropping out of school”. […]*
*P09: “A blesser will impregnate you, and then leave you and not take care of you or the child”. (Khayelitsha, Group 5, females)*

In addition, participants felt that these unequal power relations could result in emotional and physical abuse. A few male and female respondents noted that they could also be at risk of losing their lives when in these relationships.

*P02: “He will want you to sleep with him and do things that you do not want to do. […] Like sleeping with them or maybe even rape you”. […]*
*P07: “You get sex from blessers; it is money and sex. That person is older than you and he will use you, and you will end up losing your dignity”. (Khayelitsha, Group 6, females)*
*P04: “They lose their value and then they are abused by these blessers, like sexual and physical abuse”. [….]*
*P01: “And they are trapped in those relationships, because you cannot leave the blesser”. (Tembisa, Group 3, males)*
*P05: “A sugar mama will control you and when you want out, she will come to you house”. […]*
*P09: “Or get people to kill you, they would say ‘now that you have used my money you do not want me anymore’; and she will get people to kill you”. […]*
*P10: “A man might find out that you are dating his wife and shoot you”. (Khayelitsha, Group 7, males)*

### Health seeking behaviour and experiences

For general health related issues, participants had attended a range of healthcare facilities, including clinics, doctors, private pharmacies and hospitals. In addition, a few participants preferred to rely on traditional healers, traditional medicines, or home remedies for general health issues.

Female participants were asked if they accessed SRH services. Although some noted that they were not sexually active, and did not access SRH services, others went for HIV testing even if they believed they were not at risk of acquiring it from sexual activity.

*F: “You haven’t accessed them [SRH services], and then why? Any reasons?”*
*P: “I don’t prevent because I’m not sexually active and I don’t do abortion”. (Soweto, Group 2, females)*
*P07: “I like testing for HIV because I learnt that we don’t actually, we don’t actually get HIV from umm having sex only but there are different ways to get HIV”. (Tembisa, Group 2, females)*

A few females (mainly from Khayelitsha and Tembisa) discussed that they had accessed family planning, HIV and/or STI testing services from healthcare facilities.

*P02: “Yes, I have went to the clinic, but not with my boyfriend. […] to test for HIV, STIs and all the other illnesses; I also go to get the injection (contraceptive)”. […]*
*P01: “I go to the clinic after every three months to check whether there are any illnesses that I have contracted to make sure that I am still fine”. […]*
*P07: “I go to the clinic to check for HIV, because HIV can be contracted in other ways besides through having sex. So, it is important to get checked”. (Khayelitsha, Group 6, females)*

In terms of access to HIV testing services with their partners, males in one group made reference to the following:

*F: “OK, so when you fall ill where do you go and what do you do?” [...] *
*P09: “I go to the clinic or I go with my baby (girlfriend) and we go to the clinic”. […]*
*P10: “I tell my girlfriend that I am not feeling well, and we both go and test (for HIV)”. (Khayelitsha, Group 7, males)*

***Access to services: Experiences and barriers***. Some participants described positive experiences accessing healthcare services from various facilities – sometimes because services were paid for, and other times they felt the healthcare providers were non-judgmental and caring.

*F: “OK. So, what was the treatment like at the clinic?” […]* *P04: “They treated me well, they gave me special treatment. Like they spoke well with me and gave me medications”. [….]* *P06: “The nurse was laughing with me. Yes, she was friendly”. [….]* *P05: “I also received good treatment because I paid, I used my money”*. *F: “Oh, so money talks?”* *P05: “Yes, it was my money”. […]* *P03: “When I arrived, they provided a quick service because they could see that I am a learner”. (Soweto, Group 1, males)*
*P08: “Like me I go to [name of clinic], the reason I’m going there is because they are understanding, they know what’s needed, they understand situations of teenagers and they are not judgemental. They won’t be judging me of why am I doing certain things because I’m still a child, they just give you whatever you need”. (Tembisa, Group 3, females)*

Others described mixed experiences attending healthcare facilities.

*P1: “Close to where I stay…when I go with my mom, they’re fine, but when I’m alone they give me problems it’s not easy to get a service”. (Khayelitsha, Group 6, males)*
*P10: “I prefer going to [name of clinic] because I feel more comfortable with the nurse because they know how to speak with young people. Unlike [name of other clinic], when you go to prevent, they will say “you are still young, why you are having sex?”, yah things like that”. (Tembisa, Group 3, females)*

The majority of participants however, had negative experiences, resulting in barriers to them accessing healthcare services. Some females from all three townships described how the attitudes of healthcare providers actually prevented them from accessing services they may need.

*F: “Who swears at you?”* *A few people in the group: “The nurses!”* *PID unknown: “They embarrass you in front of people, and that time there would be a lot of people”. (Khayelitsha, Group 2, males)*
*P02: “Yes, the nurses like to shout if you are young and you came alone, they shout. Sometimes they ignore you, and the line does not move. So, you stay there for a long time”. (Khayelitsha, Group 1, females)*
*F: “Which services do you access at health facilities?” […]* *P06: “Nothing because the nurses are so rude and they humiliate you”. (Soweto, Group 1, females)*

### Contraceptive method preferences, perceptions, and decision-making behaviour

***Contraceptive method preferences.*** Preferences for contraceptive methods were closely related to the perceived advantages and disadvantages of the different methods. Female participants were asked about their own and their peers’ preferred contraceptive methods. Responses were general, with many females talking about community preferences rather than their own preferences for contraceptive methods.

Participants from all groups described a preference for injectable contraceptives. This was followed by the implant, and next was an equal preference for condoms and oral pills. This was followed by the IUD and the patch and abstinence. There was minimal awareness of emergency contraception.

***Perceived benefits of different contraceptive methods.*** The perceived benefits of various contraceptive methods were closely linked to method preferences. Females favoured long-acting methods (implant and IUD) and/or injectable contraceptives because using these methods did not require frequent visits to a healthcare facility.

*P04: “The implant. People who are in school cannot frequently go to the clinic. So, the implant is fine for females”. (Khayelitsha, Group 7, females)*
*P16: “The copper one (IUD) because you can stay with it I think for 5 years and you don’t have to go back and forth (frequently) to the clinic. It stays there and you forget about it”. (Soweto, Group 1, females)*
*P07: “Injection because you do not have to keep going back to the clinic. You go once, and then return after three months”. (Khayelitsha, Group 5, females)*

An added advantage of the injectable contraceptive was that one didn’t need to remember to take it daily.

*P11: “I think the injection (is preferred) because you can forget to take your pills and fall pregnant”. (Soweto, Group 1, females)*

One participant felt that the lack of menses (and no period pains) experienced as a side effect of the implant was beneficial.

*P03: “[…] the implant because I don’t want to go on my periods, so my mother says that it depends on your body and the side effects are not the same, it is only for people who have grown and don’t want to have periods”*. *F: “Okay, why don’t you want periods?”* *P03: “Period pains eish (sigh)”. (Soweto, Group 3, females)*

Condoms were preferred by a few females who suggested that they were easier to access, and that they did not need to go to healthcare facilities (where they were not treated well) to access them. In addition, the choice of flavours was described as an advantage of condom use.

*PID unknown: “Most of them use condoms because like they are ashamed to go to the hospital to get injection, so they think umm condom is the easy way, yah”. (Tembisa, Group 2, females)*
*F: “Why do you prefer the condom?”* *P06: “Because you can change different flavours”. (Soweto, Group 1, females)*

Oral contraceptives were viewed as beneficial by only one participant, who preferred them because one can control their daily use.

*P03: “They prefer pills because they can control them daily, if they want to prevent, they can go off it and on it”. (Soweto, Group 3, females)*

***Perceived barriers to contraceptive use.*** Some of the described barriers to contraceptive use were linked to method side effects and others to myths and misconceptions about contraceptive methods.

The most common disadvantage of a contraceptive method, described by adolescent females, was that people could forget to take their oral contraceptive pill. In addition, participants from one group from Soweto mentioned that if emergency contraception was not taken timeously, it would be ineffective.

*P03: “Yes, the pill is not safe because you can forget to take it and the coaches told us that if you forget to take it you can fall pregnant”. (Khayelitsha, Group 2, females)*
*P02: “The morning after pill might not work because you have to think about when you take it like a certain amount of time I think 72 hours must not pass before you take it so if you’re working shifts it might be a problem. Same goes for the pill. You must be careful if you work shifts”. (Soweto, Group 1, females)*

There were concerns about the injectable – some noted that people may fear injections, others suggested that side effects, such as weight gain associated with use, were a concern.

*PID unknown: “Like some are scared of injection”. (Tembisa, Group 3, females)*
*PID unknown: “The three months (injectable contraceptive) makes you fat”. (Tembisa, Group 3, females)*

The side effects related to menses - excessive bleeding or amenorrhea, were viewed as disadvantages of the injectable contraceptive or oral pill by some. One participant from Soweto had concerns that lack of menses when using an injectable contraceptive may impact on growth and development.

*P09: “Eish, like coach, my sister was taking the pill and she stopped having her period for two months. When it came again it lasted for 20 days”. (Soweto, Group 1, females)*
*P04: “They say when you inject you bleed a lot”. (Soweto, Group 3, females)*
*P03: “[…] because with injection you don’t go on your periods for a long time so there is a fear that I injected this and when I develop and grow over the years whilst I am only 13 years so what will happen”. (Soweto, Group 3, females)*

Furthermore, females in one group from Tembisa also raised concerns of future infertility from using the injectable or oral contraceptive.

*P09: “I think some of them they have bad, uh they have disadvantage because like maybe for who are getting injected or those taking pills it could stop them from making children in future. […] Yah so when they face the problem like they won’t have children because of the things they were using”. (Tembisa, Group 2, females)*

The implant was perceived to be dangerous to use by some participants from Khayelitsha – with the rumour that it could be stolen from their arms, as it was believed that people use the implant for drugs.

*PID unknown: “The skollies (bad people) smoke implant. So, they mug you off it”*. *PID unknown: “They pull it out of your arm because it is visible, so they cut you and then remove it from your arm”. (Khayelitsha, Group 2, females)*

In addition, females from a group in Soweto had concerns that the implant was not an effective contraceptive method (possibly related to drug interactions), and had false beliefs that it could rust inside one’s arm.

*P01: “Ahhh the Implanon (implant) is not good because people fall pregnant while having the implanon in their arms and now when you’re pregnant you must get it removed”*. *F: “So people fall pregnant even with the implanon inserted?”* *Group: “Yes…” […]* *P05: “And they say it can actually begin to rust inside your arm and you develop a wound”. (Soweto, Group 1, females)*

The IUD was believed, by one participant from Khayelitsha, to take up space inside the vagina.

*F: “OK, and then so the methods that females do not like which ones are those?”* *PID unknown: “Loop (IUD) because it does not leave any space”. (Khayelitsha, Group 2, females)*

Condom use was also described as a challenge by participants from Soweto who said that people prefer condomless sex.

*PID unknown: “Others don’t want a condom they want skin to skin”. (Soweto, Group 3, females)*

***Contraceptive decision making.*** Females were asked who makes the decision for young females to start using contraception. The majority of them noted that it was either their parents (usually specifying their mother), or themselves that made the decision.

*P01: “It is me; because I still want to study, and finish school. I do not want to be like other females here at school that fall pregnant, and never return to school after giving birth”*. *F: “OK, so it is about protecting yourself and securing your future; anyone else? Who makes the decision?” […]* *P08: “My mother because she will say, ‘my child you are old now’; she will say something like, ‘you have started seeing, now so you must go to the clinic’”*. *F: “OK, so a parent makes that decision; but why?”* *P08: “Because she does not want to get embarrassed when you fall pregnant. She would rather send you to the clinic even if she does not know whether you are having sex or not. She just wants to make sure that it (pregnancy) does not happen”. (Khayelitsha, Group 5, females)*

A few suggested that their boyfriends/partners encouraged contraceptive method use, and that they made the decision together. One participant suggested that some partners force females to use contraception.

*P09: “But others they like to use them (contraceptives) is because like they, people they date encourage them to use these because they are not ready to have children and so on”. (Tembisa, Group 2, females)*
*P03: “It is the two of us; me and my partner”. (Khayelitsha, Group 7, females)*
*P05: “[T]hey were being forced by their partner’s maybe because the partner won’t be able to support her”. (Tembisa, Group 2, females)*

Another participant suggested that friends influence their decisions to use contraceptive methods.

*P09: “Some they are being talked by their friends, is better if like you wanna have sex use a condom or to prevent, because like they don’t wanna get pregnant at a younger age, what will your friends say? Yah”. (Tembisa, Group 2, females)*

## Discussion

Adolescents in this study demonstrated that they had acquired SRH knowledge during the GAP Year intervention, including information on SRHR, risk reduction and where to obtain services. They also learnt about HIV and STI acquisition and prevention, dual protection, as well as about various contraceptive methods.

Our findings confirm that transactional sexual and age disparate relationships were occurring in these communities. As has been reported elsewhere, adolescents engage in these relationships for material gain – sometimes for survival, especially in poor communities, and sometimes for improved lifestyles ^19, 20^. In this study, it was also reported that in some cases adolescents engage in these relationships to seek love and affirmation which they do not get at home. Despite the perceived benefits of these relationships, adolescents in the FGDs had an awareness that they could also have negative consequences, where power imbalances result in gender inequalities, and the potential for risk of GBV. Lack of power was also seen to reduce capacity to negotiate safer sex practices. These negative factors can result in increased sexual health risk - including pregnancies, HIV and STI transmission. However, despite the perceived risks of these transactional relationships, participants described some level of acceptability and support, also noted elsewhere ^66^, especially in the context of poverty, and where such relationships lead to improved living conditions for an individual and their family ^67^. The awareness of age disparate relationships was not explored, and this is of concern, given the association with increased risk of HIV ^8^.

Adolescent access to SRH services is a challenge globally, and more specifically in sub-Saharan Africa ^28– 30^. Similarly, most adolescents in this study reported barriers to accessing SRH services, largely related to provider attitudes and fear of stigma. A few even chose not to access these services because of barriers, putting them at increased risk of unintended pregnancies, STIs and HIV acquisition. Reassuringly, some participants described positive experiences accessing SRH services at healthcare facilities, largely related to strategies they had developed to improve their experiences, for example choosing to attend a healthcare facility with a reputation for being youth friendly, or accessing services with a parent, to avoid judgmental attitudes. A few participants described making use of traditional healers or home remedies for health-related concerns.

Preference, choice and uptake of contraceptive methods has been linked to access to and knowledge about methods ^47, 68^. Perceived benefits and challenges with contraceptive use are impacted on by personal situations and understandings of use, further compounded by myths and misinformation around particular methods. The GAP Year intervention provided information on male and female condoms, the injection, emergency contraception, the implant and the IUD. Participants demonstrated an understanding of the benefits of long-acting reversible contraception (LARC), and also mentioned emergency contraception.

The most preferred contraceptive method in this study was the injectable, reported in other research in South Africa and sub-Saharan Africa ^39, 41, 69^. Young females in this study also had positive perceptions about LARCs (including both the IUD and implant). The reasons for this were similar to those associated with using injectable contraceptives – the fact that they do not need to remember to use it daily, and also because of the less frequent need to access healthcare services when using these methods, also found in research with implant users ^44^. This is important as South Africa’s method mix is still dominated by injectables, and awareness and use of long acting methods is low ^43^. The promotion and awareness of LARCs is emphasised in the DOH guidelines ^70^, as well as the promotion of informed choice, including implants and IUDs. LARCs have a higher efficacy and continuation rate ^43, 71^ and are suitable for use by adolescents ^72^. Adolescent preferences for LARCs and injectables could also have implications for long-acting HIV prevention methods, including injectable PrEP and vaginal rings – where less frequent access to healthcare services is required. Similarly, several participants noted a challenge with the oral contraceptive pill in terms of remembering to take it daily, and this too needs to be taken into account with daily oral PrEP. Individual product attributes should be considered together with these perceived advantages when designing information sessions and promotional activities for long-acting HIV prevention products.

Individuals also expressed concerns about some methods, and many of these were related to side effects (real or perceived) and misconceptions, especially regarding the implant, in particular the belief that it is stolen from the arm and used for drugs, the misconception that it can rust in one’s arm, stunted growth, infertility, and low efficacy. Such concerns and misconceptions have been noted elsewhere ^44^ and can discredit the use of methods. It is therefore important that rumours and misconceptions are addressed in order that they do not undermine contraceptive programmes and method choice ^46, 73^.

Decision making about contraceptive method uptake and choice was largely made together with parents (mothers in particular), or by the adolescent females themselves. This could be linked to the fact that the GAP Year intervention engaged parents, encouraging parent-child communication. Given that evidence shows that parents experience challenges discussing SRH issues with their children ^67^, more needs to be done in future programmes focussing on parents/guardians and how to communicate effectively about sex. Only a few young females reported that their partners played a role in their contraceptive choice and use, this differs from research which shows that male partners influence contraceptive uptake and use in other areas in South Africa ^74^.

The GAP Year intervention provided these learners with important SRHR information and strategies to reduce sexual risk. One benefit that participants noted was that they learnt how to use both male and female condoms. This was covered in the Year 2 curriculum but then reinforced during the graduation events, where a local adolescent SRH organisation repeated these practical demonstrations. The importance of not only promoting condom use and dual protection in HIV/SRH prevention programmes, but also providing practical guidance on the use thereof is important, and sometimes neglected ^75, 76^.

There are several recommendations that can be made based on the findings from this study. Adolescents need to be provided with education and information on SRH rights and contraceptive methods, relative benefits of each, mechanisms of action, and side effects, as well as on service delivery points. Furthermore, these programmes need to address myths and misinformation and replace them with facts to ensure informed decision making and choice, and to strengthen uptake and correct method use. Increased dose of programmes/interventions and reinforcement of information is necessary to ensure that myths and misinformation are not perpetuated. Practical demonstration and discussions about condom use in the context of young people’s sexual lives also needs to be included. This can be done via school-based education programmes ^28^ like the GAP Year intervention, as well as through healthcare provider training and education to address knowledge gaps and judgmental attitudes ^10^. In order to bring about behaviour change, particularly in light of safe sex practices, risk reduction and health seeking behaviour, we need programmes like the GAP Year intervention, which complement the current CSE curriculum in schools, which reinforce health-seeking behaviour, and use of peer mentors. Healthcare training strategies can be reconsidered, training smaller groups, on focussed topics, with supportive supervision and problem-solving strategies ^30^. Furthermore, creating welcoming healthcare facilities, enabling confidential and youth friendly service provision, should improve service uptake ^10, 77^. In addition, providing adolescent health services at a community level, at health education events, could increase uptake and acceptability of services. Cash transfer systems and other structural interventions have been demonstrated to reduce HIV risk behaviours such as transactional sex in young girls, and should be further considered ^78, 79^. Finally, as has been suggested elsewhere ^48^, using evidence from studies on adolescents is important for informing future policies and programmes that are effective and relevant to adolescents.

### Strengths and limitations

Whilst this qualitative study is not an evaluation of the broader GAP Year Trial (trial results to be published elsewhere), the FGDs provide insight to the SRH knowledge gained as well gaps in information of some learners who participated in the GAP Year intervention. Male and female learners who had participated in the programme were asked different questions during the FGD and so comparison between groups, with regards to sex-specific knowledge and experiences was not always possible. However, there is sufficient data to get a sense of the different issues encountered by each group, and where differences have been noted, these have been highlighted.

There are also potentially a few gaps in the information gathered. Data regarding non-participation was not collected during the recruitment phase for the FGDs. Participants were not asked any questions about LGBTQI (lesbian, gay, bisexual, transgender, queer and intersex) relationships and their access to SRH services, so any challenges or experiences for these adolescent groups have not been explored. Furthermore, although participants were asked about transactional sex relationships, there were no probes further exploring age disparate relationships and the impact of this on SRHR.

There is a high portion of missing data in the demographic data presented in this manuscript, resulting in a skewed representation of male participant demographic responses. The FGDs were evenly split according to male and female groups, and therefore it is likely that much of the missing baseline data would have been female participant responses. Although baseline demographic data were extracted for the FGD participants, they are not directly linked to FGD participant responses. Furthermore, they were collected two years prior to the FGDs and therefore it is possible that some of the socio-demographic information could have changed over time. For example, the proportion of the FGD participants who were sexually active at baseline, may have changed by the time of the FGD. 

Since FGD participant responses are not directly linked to the demographic data, and because of potential changes over time, it is not possible to determine which FGD participants had actually engaged in sexual activity. For this reason, some FGD participants may not have actually accessed SRH, HIV and family planning services, and therefore may have responded to questions based on hearsay, or social desirability bias.

## Conclusions

There is a paucity of literature exploring SRH and contraceptive knowledge and perceptions of younger adolescents in South Africa. This research has been important in providing information on the SRHR knowledge and perceptions of a group of adolescent learners. Furthermore, these learners demonstrated that they had acquired knowledge during the GAP Year intervention, highlighting that an afterschool model of education provision within a school setting is a feasible model for provision of SRHR education to learners.

Findings from this study highlight what can be included in future interventions. Youth focussed interventions should focus on information provision to facilitate understanding and informed decision making. For example, the mechanism of action of contraceptives should be described to address and dispel myths around method use. Full information about different methods available, including LARCs, are important for method choice. In addition, condom promotion should provide practical information and instructions on condom use. The provision of information enables informed choice, decision-making, and method use, and could facilitate uptake of contraceptives. Furthermore, more work needs to be done to limit barriers to accessing healthcare services and to facilitate more youth friendly facilities, including reconsidering healthcare training strategies. Finally, discussions about HIV/SRH prevention need to integrate together with messages about choice and informed decision-making relating to both HIV prevention and contraceptive options.

## Data availability

### Underlying data

The transcripts have been deidentified; however, due to the nature of the topic, they are not openly available. The transcripts can be accessed by emailing the corresponding author ( mpleaner@mweb.co.za). A valid request, which will be considered by the authors, is required to access the transcripts.

Harvard Dataverse: GAP Year_learner SRH qualitative manuscript. https://doi.org/10.7910/DVN/VRSCQD ^63^.

This project contains the following underlying data:

- FGD participant demographics_final.xlsx

### Extended data

Harvard Dataverse: GAP Year_learner SRH qualitative manuscript. https://doi.org/10.7910/DVN/VRSCQD ^63^.

This project contains the following extended data:

- GAP Year Boys FGD Guide
- GAP Year Girls FGD Guide
- COREQ checklist

Harvard dataverse: GAP Year_Violence REDCap and ACASI data. https://doi.org/10.7910/DVN/AHHWNL ^61^.

This project contains the following extended data:

- GAP Year Boys ACASI Survey Questionnaire
- GAP Year Girls ACASI Survey Questionnaire
- GAP Year Boys REDCap Survey Questionnaire
- GAP Year Boys REDCap Survey Questionnaire
- GAPYear_REDCap Codebook.pdf
- GAP Year_ACASI Boys Survey Codebook.pdf
- GAP Year_ACASI Girls Survey Codebook.pdf

Data are available under the terms of the Creative Commons Zero "No rights reserved" data waiver (CC0 1.0 Public domain dedication).
