# Supplementary material for: Sexual and reproductive health and rights knowledge, perceptions, and experiences of adolescent learners from three South African townships: qualitative findings from the Girls Achieve Power (GAP Year) Trial
Source: Gates Open Res. 2022 Nov 30;6:60. Originally published 2022 May 3. [Version 2] doi: 10.12688/gatesopenres.13588.2 (PMC10220247; doi:10.12688/gatesopenres.13588.2)
Supplement: Peer review history

# Peer review: Reviewer response for version 2

**DOI:** 10.21956/gatesopenres.15523.r33213

**Published:** 2023-05-26

**Authors:** Jane Harries

**Affiliations:** 1 Division of Social and Behavioural Sciences, School of Public Health, University of Cape Town, Cape Town, South Africa

This is a well written article on an important topic. I suggest a copy edit as I picked up a few typos.

 My comments are meant to strengthen the article and many of the issues have already been addressed by the previous reviewer.

**Abstract**: “NVivo was used for data analysis” - please change to 'NVivo was used for data management'. Software packages can assist in data management and sorting but can not analyze qualitative data.

**Myths**: The term "myths" throughout the manuscript in relation to contraceptive use is somewhat problematic and is not clearly defined. Local beliefs need to be acknowledged and examined and too often local beliefs are not interrogated and loosely defined as myths.  These so called myths are not really discussed apart from related to implants being removed – “belief that it is stolen from the arm and used for drugs” this has been reported as real in SA and is not surprising with high levels of violence – see reference Harries *et al.* (2019 ^1^).

**Typos**: “I leant that when sleeping with a girl you might get a disease without using a condom … " replace 'leant' with 'learnt'. Pg.13: "the viability of improving access to contraception at schools needs to be further researched and explored." Should be "The viability ... ".

**Results:** Results section could be more synthesized as there are many quotes per sub heading that are saying similar things. The narrative gets lost with similar quotes on one topic area. This is up to the authors to decide.

Is the work clearly and accurately presented and does it cite the current literature?

Yes

If applicable, is the statistical analysis and its interpretation appropriate?

Not applicable

Are all the source data underlying the results available to ensure full reproducibility?

No source data required

Is the study design appropriate and is the work technically sound?

Yes

Are the conclusions drawn adequately supported by the results?

Yes

Are sufficient details of methods and analysis provided to allow replication by others?

Yes

Reviewer Expertise:

Qualitative research; sexual and reproductive health and rights

I confirm that I have read this submission and believe that I have an appropriate level of expertise to confirm that it is of an acceptable scientific standard.

## References

[1] : A multidimensional approach to inform family planning needs, preferences and behaviours amongst women in South Africa through body mapping. *Reprod Health* .2019;16(1) : 10.1186/s12978-019-0830-6 159 10.1186/s12978-019-0830-6 31694648PMC6836666

# Peer review: Reviewer response for version 2

**DOI:** 10.21956/gatesopenres.15523.r32783

**Published:** 2022-12-07

**Authors:** Zoe Duby

**Affiliations:** 1 Division of Social and Behavioural Sciences in the School of Public Health and Family Medicine, University of Cape Town, Cape Town, South Africa; 2 Health Systems Research Unit, South African Medical Research Council, Cape Town, South Africa

I am satisfied with the revisions, and have no further comments.

Is the work clearly and accurately presented and does it cite the current literature?

Partly

If applicable, is the statistical analysis and its interpretation appropriate?

I cannot comment. A qualified statistician is required.

Are all the source data underlying the results available to ensure full reproducibility?

Yes

Is the study design appropriate and is the work technically sound?

Yes

Are the conclusions drawn adequately supported by the results?

Yes

Are sufficient details of methods and analysis provided to allow replication by others?

Yes

Reviewer Expertise:

Sexual and reproductive health, HIV, South Africa, Qualitative research, adolescents and young people, women, gender, sexual decision making, sexual risk behaviour, HIV prevention

I confirm that I have read this submission and believe that I have an appropriate level of expertise to confirm that it is of an acceptable scientific standard.

# Peer review: Reviewer response for version 1

**DOI:** 10.21956/gatesopenres.14858.r32008

**Published:** 2022-07-05

**Authors:** Zoe Duby

**Affiliations:** 1 Division of Social and Behavioural Sciences in the School of Public Health and Family Medicine, University of Cape Town, Cape Town, South Africa; 2 Health Systems Research Unit, South African Medical Research Council, Cape Town, South Africa

Overall the paper is very well written, and comes across as comprehensively contextualised and well-thought out. I do have a few comments for consideration, which I believe would strengthen the paper.

 General comments:

1. Discussion around sexuality seems to be missing from the paper - other than a statement in the limitations section that states that FGDs did not include questions about LGBTIQ relationships. It would add depth to include discussion of how sexuality is situated in young people’s perceptions and experiences relating to SRH and sexual behaviour. How was sexuality covered in the GAP Year curriculum and materials?
2. There could be a more in-depth discussion of the sociocultural framing of transactional sex and relationships in these communities, that normalise it, and in which people consider it a viable source of income. Also in terms of sociocultural informed gendered expectations of exchange.
  Reference to consider: Duby *et al.* (2021 ^1^).
3. Knowledge about condoms and HIV does not always translate to being able to, or wanting to, enact “safer” sex practices. What about discussion of pleasure, intimacy, and how these might impact. Understanding the complexity of actually practicing “safer sex” requires unpacking the complex factors that influence a behaviour such as condom use.
  Reference to consider: Duby *et al. *(2021 ^2^).
4. Another South African context reference to consider is: Jonas *et al.* (2020 ^3^).
5. Was there any discussion of how parents are/can be included in young people’s sexuality education? Discussion of the role of peers/family/parents may strengthen the discussion.

Specific comments:

1. Abstract Methods section: “Overall, 26 focus group discussions (FGDs): 13 male and 13 female learner groups, purposively selected from schools, after the intervention (2 years after baseline data collection)” – word missing from sentence?

Methods section:

1. The term ‘risky behaviours’ should be problematised, as framing specific behaviours as inherently risky. The authors should read discussion around the “discourse of risk” in public health literature – particularly discussions around “Youth and their Risk-taking”.
2. Check acronyms are consistent – in one sentence appears “FDGs”.
3. How was participants’ gender assigned, for allocation into “male/female” FGDs? Was this self-identified gender?
4. What about sexuality/sexual orientation of participants, was this taken into account?
5. Who translated the FGDs? Was there some kind of quality/accuracy review of translations to ensure interpretation was accurate?

Is the work clearly and accurately presented and does it cite the current literature?

Partly

If applicable, is the statistical analysis and its interpretation appropriate?

I cannot comment. A qualified statistician is required.

Are all the source data underlying the results available to ensure full reproducibility?

Yes

Is the study design appropriate and is the work technically sound?

Yes

Are the conclusions drawn adequately supported by the results?

Yes

Are sufficient details of methods and analysis provided to allow replication by others?

Yes

Reviewer Expertise:

Sexual and reproductive health, HIV, South Africa, Qualitative research, adolescents and young people, women, gender, sexual decision making, sexual risk behaviour, HIV prevention

I confirm that I have read this submission and believe that I have an appropriate level of expertise to confirm that it is of an acceptable scientific standard, however I have significant reservations, as outlined above.

## References

[1] : From Survival to Glamour: Motivations for Engaging in Transactional Sex and Relationships Among Adolescent Girls and Young Women in South Africa. *AIDS Behav* .2021;25(10) : 10.1007/s10461-021-03291-z 3238-3254 10.1007/s10461-021-03291-z 33950338PMC8416808

[2] : “Condoms Are Boring”: Navigating Relationship Dynamics, Gendered Power, and Motivations for Condomless Sex Amongst Adolescents and Young People in South Africa. *International Journal of Sexual Health* .2021;33(1) : 10.1080/19317611.2020.1851334 40-57 10.1080/19317611.2020.1851334 PMC1080780538596471

[3] : Perceptions of contraception services among recipients of a combination HIV-prevention interventions for adolescent girls and young women in South Africa: a qualitative study. *Reprod Health* .2020;17(1) : 10.1186/s12978-020-00970-3 122 10.1186/s12978-020-00970-3 32795366PMC7427945

# Response

**Published:** 2022-11-21

**Authors:** Alison Kutywayo

**Affiliations:** Wits Reproductive Health and HIV Institute, South Africa

The authors thank the reviewer for their extensive review. We have made the following changes in response to their feedback: 

 Discussion around sexuality seems to be missing from the paper - other than a statement in the limitations section that states that FGDs did not include questions about LGBTIQ relationships. It would add depth to include discussion of how sexuality is situated in young people’s perceptions and experiences relating to SRH and sexual behaviour. How was sexuality covered in the GAP Year curriculum and materials?

- Added sections and additional references throughout the manuscript to address this query, especially in the Introduction and the Discussion

There could be a more in-depth discussion of the sociocultural framing of transactional sex and relationships in these communities, that normalise it, and in which people consider it a viable source of income. Also in terms of sociocultural informed gendered expectations of exchange.  Reference to consider: Duby *et al.* (2021 ^1^ ).

- We agree with this comment and have added the Duby et al. reference at the end of this sentence in the Introduction (paragraph 3) as well as to the Discussion (paragraph 2)

Knowledge about condoms and HIV does not always translate to being able to, or wanting to, enact “safer” sex practices. What about discussion of pleasure, intimacy, and how these might impact. Understanding the complexity of actually practicing “safer sex” requires unpacking the complex factors that influence a behaviour such as condom use.

 Reference to consider: Duby *et al. *(2021 ^2^ ).

- Agree - added a sentence on this in Discussion (paragraph 8) as well as this reference 

Another South African context reference to consider is: Jonas *et al.* (2020 ^3^ ).

- Have added this reference in several instances: In the Introduction (paragraph 4) and Discussion (3rd last paragraph)

Was there any discussion of how parents are/can be included in young people’s sexuality education? Discussion of the role of peers/family/parents may strengthen the discussion

- Discussion: paragraph 7 refers to parents/guardians.

Abstract Methods section: “Overall, 26 focus group discussions (FGDs): 13 male and 13 female learner groups, purposively selected from schools, after the intervention (2 years after baseline data collection)” – word missing from sentence?

- Word has been added to this sentence for meaning.

Methods; The term ‘risky behaviours’ should be problematised, as framing specific behaviours as inherently risky. The authors should read discussion around the “discourse of risk” in public health literature – particularly discussions around “Youth and their Risk-taking”.

- Term has been amended to: ‘behaviours that put them at increased risk’.

Methods:  Check acronyms are consistent – in one sentence appears “FDGs”.

- Amended.

Methods:  How was participants’ gender assigned, for allocation into “male/female” FGDs? Was this self-identified gender?

- Added: 'Participants were allocated to a male/female group, based on their self-identified sex, indicated on their baseline survey.'

Methods:  What about sexuality/sexual orientation of participants, was this taken into account?

- Sexual orientation of participants was not considered in the allocation to a FGD.

Methods:  Who translated the FGDs? Was there some kind of quality/accuracy review of translations to ensure interpretation was accurate?

- This has now been included.
